# New insights into salvianolic acid A action: Regulation of the TXNIP/NLRP3 and TXNIP/ChREBP pathways ameliorates HFD-induced NAFLD in rats

**DOI:** 10.1038/srep28734

**Published:** 2016-06-27

**Authors:** Chunchun Ding, Yan Zhao, Xue Shi, Ning Zhang, Guo Zu, Zhenlu Li, Junjun Zhou, Dongyan Gao, Li Lv, Xiaofeng Tian, Jihong Yao

**Affiliations:** 1 Department of Pharmacology, Dalian Medical University, Dalian, 116044, China; 2 Department of General Surgery, Second Affiliated Hospital of Dalian Medical University, Dalian, 116023, China

## Abstract

Salvianolic acid A (SalA), one of the most efficacious polyphenol compounds extracted from Radix *Salvia miltiorrhiza* (Danshen), has been shown to possess many potential pharmacological activities. This study aimed to investigate whether SalA has hepatoprotective effects against high-fat diet (HFD)-induced non-alcoholic fatty liver disease (NAFLD) and to further explore the mechanism underlying this process. SalA treatment significantly attenuated HFD-induced obesity and liver injury, and markedly decreased lipid accumulation in HFD-fed rat livers. Moreover, SalA treatment ameliorated HFD-induced hepatic inflammation and oxidative stress by decreasing hepatotoxic levels of cytokines, suppressing the overproduction of reactive oxygen species (ROS) and methane dicarboxylic aldehyde (MDA) and preventing the decreased expression of superoxide dismutase (SOD). Importantly, SalA reversed the HFD- or palmitic acid (PA)-induced activation of the NLRP3 inflammasome, the nuclear translocation of ChREBP and the up-regulation of FAS, and these effects were accompanied by TXNIP down-regulation. However, TXNIP siRNA treatment partially abrogated the above-mentioned effects of SalA in PA-treated HepG2 cells. Together, our results demonstrated, for the first time, that SalA protects against HFD-induced NAFLD by ameliorating hepatic lipid accumulation and inflammation, and these protective effects may partially due to regulation of the TXNIP/NLRP3 and TXNIP/ChREBP pathways.

Non-alcoholic fatty liver disease (NAFLD) has a worldwide distribution and is now considered the leading cause of chronic liver disease. It has been suggested that NAFLD spans a spectrum of disease from hepatic steatosis to nonalcoholic steatohepatitis (NASH), fibrosis, cirrhosis, and hepatocellular carcinoma (HCC)^1^,^2^. Nonalcoholic steatosis is mainly characterized by intracellular triglyceride (TG) and total cholesterol (TC) accumulation, which results from an imbalance between increased lipid availability via circulating lipid uptake or *de novo* lipogenesis and decreased lipid disposal, mainly through free fatty acid (FFA) oxidation, and it has been considered the first hit in the pathogenesis of NAFLD. High levels of plasma TG, TC and saturated fatty acids contribute to metabolic syndrome-associated inflammation and the secretion of pro-inflammatory cytokines, which lead to oxidative stress resulting from excessive reactive oxygen species (ROS) production, constituting the second hit in the pathogenesis of NAFLD^3^,^4^,^5^,^6^. Thus, a better understanding of the steps involved in regulating lipid accumulation, inflammation and oxidative stress might provide a new therapeutic strategy for NAFLD prevention and treatment^7^,^8^. Furthermore, with the exception of management through diet and exercise^9^,^10^, the effective approaches available for controlling the onset and progression of NAFLD remain limited.

*Salvia miltiorrhiza* Bunge (Danshen/Red sage, SM), a well-known traditional herbal medicine, has been widely used in China for centuries. Salvianolic acid A (SalA) is one of the most efficacious phenolic acids of seven water-soluble compounds isolated from the Radix *Salvia miltiorrhiza*^11^. Many studies have reported that SalA possesses a variety of pharmacological properties, including anti-oxidant, anti-inflammatory, anti-fibrotic and anti-carcinogenic activities^12^,^13^,^14^. Moreover, SalA demonstrated potent protective effects against hepatic fibrosis in high-fat diet (HFD)-fed type 2 diabetic rats, which was likely due to its anti-oxidative and anti-apoptotic properties^15^. In our previous studies, SalA alleviated concanavalin A (ConA)-induced acute liver injury by inhibiting apoptosis and inflammation^16^. Furthermore, recent studies by our group and others have shown that salvianolic acid B (SalB) and several other polyphenols can protect against NAFLD^17^,^18^,^19^,^20^. Despite these observations, whether SalA has a hepatoprotective effects against HFD-induced NAFLD and the underlying molecular mechanism remain unclear.

According to the “double-hit” hypothesis, inflammation and oxidative stress are vital contributors to the development of NAFLD^3^,^7^. The thioredoxin (TRX) system, one of the major mammalian antioxidant systems, plays a critical role in defense against the effects of ROS, including those of the superoxide anion (O_2_^•−^) and hydrogen peroxide (H_2_O_2_)^21^. Thioredoxin-interacting protein (TXNIP) is the endogenous regulator of the TRX system, which inhibits Trx-1 and Trx-2 in the cytosol and mitochondria, leading to robust ROS production and cellular oxidative stress^22^. It has been reported that TXNIP plays an important role in diverse cellular processes, including the regulation of cellular redox balance, inflammation, lipid metabolism and apoptosis^23^,^24^. TXNIP deficiency protects against methionine choline-deficient (MCD) diet-induced NASH in mice^25^. These findings suggest that TXNIP may be an important mediator of NAFLD, and detailed studies on the molecular mechanism governing control of TXNIP expression may reveal this protein as a potential therapeutic target in NAFLD. Furthermore, recent studies have suggested that TXNIP is a critical signaling molecule linking oxidative stress to NLRP3 inflammasome activation^19^,^26^. The NOD-like receptor (NLR) family, pyrin domain containing 3 (NLRP3) inflammasome is composed of NLRP3, apoptosis associated speck-like protein (ASC) and cysteinyl aspartate specific proteinase-1 (caspase-1). NLRP3 interacts with ASC to cleave caspase-1, leading to maturation and secretion of the pro-inflammatory cytokines interleukin (IL)-1β and IL-18, which cause inflammatory responses and tissue damage^27^,^28^,^29^. Inflammasome activators, such as cholesterol crystals, saturated fatty acids and ROS^30^,^31^, induced the dissociation of TXNIP from thioredoxin and the direct interaction between TXNIP and NLRP3, leading to the activation of the NLRP3 inflammasome^26^,^30^,^32^. Researches have begun to recognize that TXNIP/NLRP3 activation is involved in the pathogenesis of type 1 diabetes- or fructose-associated NAFLD and that some phenolic compounds may confer protective effects by targeting TXNIP/NLRP3 pathway^8^,^19^. However, whether this pathway is involved in the SalA-mediated protective effects remains unknown. Moreover, the role of the TXNIP/NLRP3 pathway in the HFD-induced healthy liver → NAFLD → NASH transition remains to be further elucidated.

Carbohydrate response element-binding protein (ChREBP), a transcriptional activator of glycolytic and lipogenic genes, has emerged as a key determinant of the control of hepatic *de novo* fatty acid synthesis under physiological conditions as well as in the context of NAFLD^4^,^33^. ChREBP-overexpressing mice fed an HFD showed greater hepatic steatosis^34^. Furthermore, ChREBP acts as a potent regulator of TXNIP expression by binding to the TXNIP promoter in INS-1 cells and human islets, leading to oxidative stress and apoptosis^35^. Interestingly, recent studies have shown that TXNIP in turn modulates ChREBP activity, resulting in the regulation of other ChREBP target genes that play important roles in glucose and lipid metabolism^36^. However, whether this cross talk mechanism between TXNIP and ChREBP contributes to the development of HFD-induced NAFLD remains unknown.

In the current study, we investigated the role of TXNIP in HFD-induced NAFLD, and we explored whether SalA down-regulates TXNIP expression during NAFLD and whether TXNIP inhibition activates protective mechanisms by inhibiting NLRP3 inflammasome activation and ChREBP nuclear translocation. Our findings provide new insights into the mechanisms underlying the pharmacological effects of SalA on NAFLD, and we expect that these findings will contribute to novel strategies for the management of NAFLD.

## Results

### SalA attenuates HFD-induced liver injury and obesity

**Figure 1:**
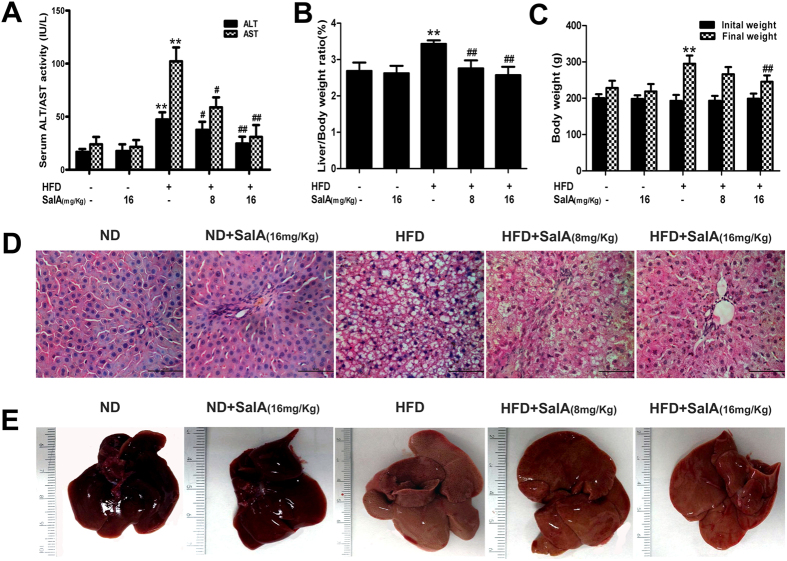
SalA alleviates HFD-induced liver injury *in vivo* and *in vitro*. Rats were fed either a normal diet (ND) or a high-fat diet (HFD) alone or in combination with SalA. (**A**) Serum levels of ALT and AST; (**B**) Liver/body weight ratio; (**C**) Body weight changes. The results represent the mean ± SD (n = 8). ^**^P < 0.01 vs. the control group; ^#^*P* < 0.05, ^##^*P* < 0.01 vs. the HFD group. (**D,E**) Representative morphological and H&E-stained images of liver sections from the following experimental groups: ND; ND + SalA (16 mg/kg); HFD; HFD + SalA (8 mg/kg) and HFD + SalA (16 mg/kg). H&E-stained sections were photographed at 400x magnification.

We first investigated whether SalA was able to protect against HFD-induced NAFLD in rats. As shown in Fig. 1(A), compared with the control group, HFD-fed rats developed liver injury, as demonstrated by higher serum levels of ALT and AST. However, these changes were significantly reversed by SalA treatment, suggesting a protective effect of SalA on HFD-induced hepatic injury. Moreover, the body weight and liver/body weight ratios in HFD-fed rats were markedly greater than those in the control group (Fig. 1(B,C)), and SalA treatment reversed this trend. The liver injury and the protective effect of SalA were further verified by liver morphologic and histological examination. As shown in Fig. 1(D,E), vehicle-treated rats exhibited no apparent abnormalities, whereas long-term HFD feeding significantly increased the liver size, changed the color of the liver and induced a large amount of lipid deposition in hepatocytes, as well as massive nuclear pleomorphism and inflammatory cell infiltration. However, SalA treatment markedly alleviated the liver injury and attenuated the hepatic lipid accumulation caused by the HFD. In addition, we mimicked the condition *in vivo* using palmitic acid (PA), as previously described^17^. SalA pretreatment increased the viability of HepG2 cells exposed to PA in a concentration-dependent manner (Fig. S1). Taken together, these results indicate that SalA confers protection against HFD-induced NAFLD.

### SalA ameliorates hepatic steatosis, inflammation and oxidative stress *in vivo* and *in vitro*

**Figure 2:**
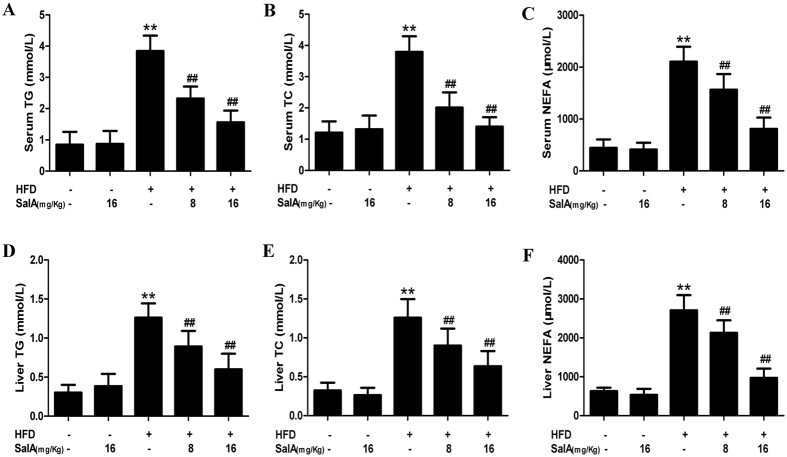
Effects of SalA on lipid accumulation in HFD-fed rats. The following biochemical indexes are shown: serum levels of TG, TC and NEFA (**A–C**) and liver levels of TG, TC and NEFA (**D,E**). The data are expressed as the mean ± SD (n = 8). ^**^P < 0.01 vs. the control group; ^##^P < 0.01 vs. the HFD group.

**Figure 3:**
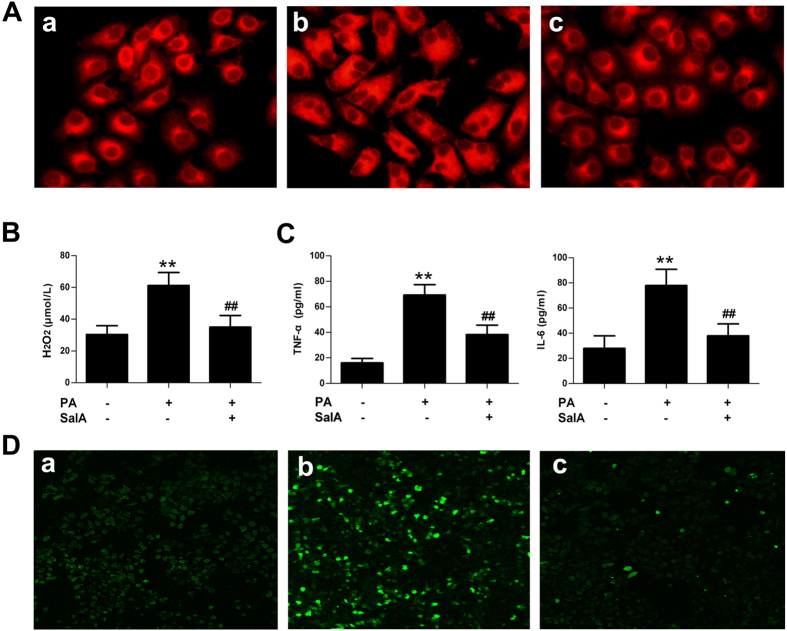
Effects of SalA on lipid accumulation, oxidative stress and inflammation in PA-treated HepG2 cells. HepG2 cells were pretreated with SalA (20 μM) for 6 h before exposure to PA (0.5 mM) for another 24 h. (**A**) Intracellular lipid accumulation was measured by Nile Red staining. (**B**) Cell supernatant H_2_O_2_ levels. (**C**) The levels of TNF-α and IL-6 in the culture medium were measured by ELISA. **(D)** Intracellular ROS levels were measured using the fluorescent probe DCFH-DA. The results are presented as the means ± SD (n = 8). ^**^P < 0.01 vs. the control group; ^##^*P* < 0.01 vs. the PA-treated group. The images were obtained using fluorescence microscopy (400×). (**A**) Control group; (**B**) PA group; (**C**) PA group pretreated with 20 μM SalA.

Hepatic steatosis, inflammation and oxidative stress are considered sequential steps in the development of NAFLD^2^. We, therefore, analyzed typical biochemical markers to further elucidate the protective effects of SalA against NAFLD. *In vivo*, our results showed that, after 10 weeks of HFD feeding, the serum and hepatic levels of TG, TC and non-esterified fatty acid (NEFA) were dramatically higher than those in the control group (Fig. 2), whereas SalA treatment abrogated these increases. *In vitro*, Nile red staining showed an increase in lipid droplets in PA-treated HepG2 cells, and the number of these droplets was decreased by SalA pretreatment (Fig. 3(A)). These results suggest a protective effect of SalA on lipid accumulation.

**Figure 4:**
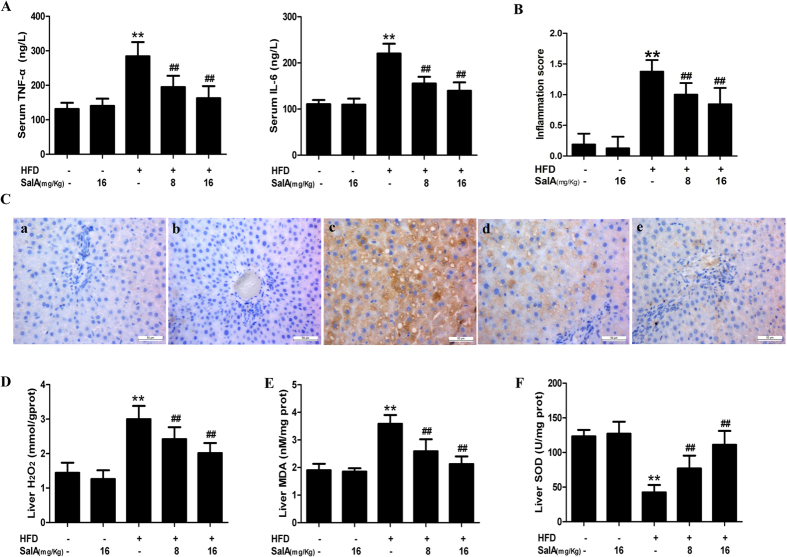
SalA protects rats against HFD-induced oxidative stress and inflammation. (**A**) ELISA-based quantification of serum levels of TNF-α and IL-6. (**B**) Histopathological assessment of the hepatic inflammation score is presented for the following experimental groups: ND; ND + SalA (16 mg/kg); HFD; HFD + SalA (8 mg/kg) and HFD + SalA (16 mg/kg). The data are expressed as the mean ± SD (n = 8). ^**^P < 0.01 vs. the control group; ^##^P < 0.01 vs. the HFD group. (**C**) Representative F4/80-stained sections of rat livers (400×). (**A**) ND group; (**B**) ND + SalA (16 mg/Kg) group; (**C**) HFD group; (**D**) HFD + SalA (8 mg/Kg) group; **(E)** HFD + SalA (16 mg/Kg) group. (**D–F**) Liver H_2_O_2_, MDA and SOD levels. The results are presented as the mean ± SD (n = 8), ^**^P < 0.01 vs. the control group; ^##^*P* < 0.01 vs. the HFD group.

Lipid accumulation might link oxidative stress to inflammation, forming a feedback loop that significantly aggravates the severity of NAFLD^37^,^38^. We therefore evaluated the state of oxidative stress and inflammation *in vivo* and *in vitro*. As shown in Fig. 4(A), compared with the control group, the HFD-fed group exhibited increased levels of pro-inflammatory cytokines, including TNF-α and IL-6. However, SalA treatment effectively decreased the TNF-α and IL-6 levels. Additionally, compared with the control group, the hepatic inflammation score was significantly elevated in HFD-fed rats (Fig. 4(B)). SalA treatment effectively alleviated these histological changes in the liver of HFD-fed rats. Macrophages have been reported to play an important role in hepatic inflammation by secreting various pro-inflammatory cytokines^39^. Thus, we investigated whether the observed reduction in pro-inflammatory cytokines could be a result of a reduced number of macrophages in the rat liver. We used an anti-F4/80 antibody to detect macrophage infiltration. As shown in Fig. 4(C), compared with the control group, immunohistochemical staining revealed a large number of F4/80-positive cells in the HFD-fed rat liver, and SalA treatment markedly decreased the number of F4/80-positive cells. Furthermore, HFD-fed rats developed serious oxidative stress, as indicated by increased hepatic levels of H_2_O_2_ and methane dicarboxylic aldehyde (MDA) and inhibition of superoxide dismutase (SOD) activity (Fig. 4(D–F)); these changes were markedly reversed by SalA treatment. In addition, compared with the control group, PA-treated HepG2 cells exhibited increased production of ROS and higher levels of H_2_O_2_, TNF-α and IL-6 in the culture supernatant (Fig. 3(B–D)); in contrast, pretreatment with SalA notably suppressed the production of ROS and H_2_O_2_ and the secretion of TNF-α and IL-6. Together, these results indicate that SalA attenuates oxidative stress and inflammation during NAFLD.

### The SalA-mediated protection against NAFLD involves TXNIP down-regulation

**Figure 5:**
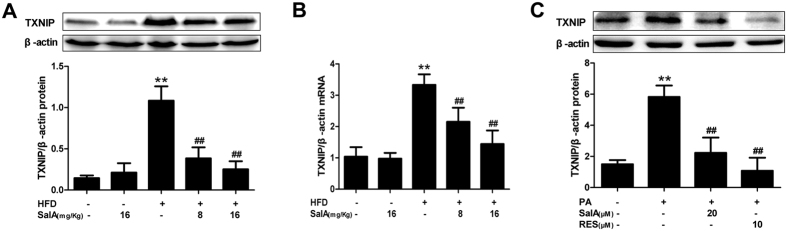
SalA reduces TXNIP overexpression in the liver of HFD-fed rats and PA-treated HepG2 cells. (**A,C**) The TXNIP protein levels in the hepatic and cellular lysates were determined by Western blotting (n = 3). (**B**) Hepatic mRNA levels of TXNIP were analyzed by RT-PCR (n = 4). Results are presented as the mean ± SD, ^**^P < 0.01 vs. the control group; ^##^*P* < 0.01 vs. the HFD group or the PA-treated group.

TXNIP has been reported to be a main regulator of the cellular reduction-oxidation balance state^21^,^22^. SalA treatment effectively attenuated hepatic oxidative stress in HFD-fed rats; thus, we investigated whether SalA confers protection against NAFLD by regulating TXNIP. The results showed that in agreement with the HFD-induced hepatic oxidative stress, the TXNIP mRNA and protein levels were significantly increased (Fig. 5(A,B)), and SalA treatment remarkably inhibited such increase. In parallel with the above *in vivo* data, PA stimulation increased TXNIP protein levels in HepG2 cells, while pretreatment with SalA or resveratrol (RES)^40^ completely abrogated this increase (Fig. 5(C)). Moreover, our small interfering RNA (siRNA) transfection results showed that compared with the control siRNA group, the SalA treatment group had remarkably lower TXNIP expression, and the SalA-mediated down-regulation of TXNIP was abrogated upon TXNIP siRNA transfection in HepG2 cells (Fig. 7(A)). These results demonstrate that SalA decreases TXNIP overexpression *in vivo* and *in vitro* and that this down-regulation may contribute to SalA-mediated protection during NAFLD.

### SalA inhibits NLRP3 inflammasome activation in a TXNIP-dependent manner

**Figure 6:**
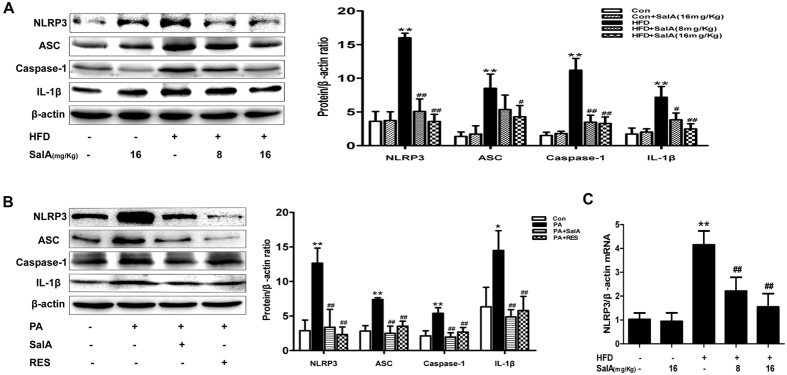
SalA inhibits NLRP3 inflammasome activation in HFD-fed rat livers and PA-treated HepG2 cells. (**A,B**) The protein levels of NLRP3, ASC, caspase-1 and IL-1β in hepatic and cellular lysates were analyzed by Western blotting in different groups, as indicated (n = 3). (**C**) Hepatic mRNA levels of NLRP3 were analyzed by RT-PCR (n = 4). Results are presented as the mean ± SD, ^*^P < 0.05, ^**^P < 0.01 vs. the control group; ^#^*P* < 0.05, ^##^*P* < 0.01 vs. the HFD group or the PA-treated group.

In accordance with the increased ROS generation and oxidative stress, serum levels of pro-inflammatory cytokines were higher in HFD-fed rats, and SalA treatment considerably reduced these increases (Fig. 3). The NLRP3 inflammasome has been reported to mediate sterile inflammation and lead to tissue damage by increasing pro-inflammatory cytokine secretion^27^. We thus investigated the relationship between SalA treatment and the activation of the NLRP3 inflammasome during NAFLD. As shown in Fig. 6(A,C), compared with the control group, HFD-fed rats showed substantial increases in the liver levels of NLRP3 mRNA and protein, as well as increased protein levels of ASC and caspase-1. However, SalA treatment effectively prevented the above-mentioned increase. *In vitro*, the PA-treated group exhibited an extraordinary increase in the expression of NLRP3, ASC and caspase-1 (Fig. 6(B)), while pretreatment with SalA or RES^41^ abrogated such an increase. Furthermore, SalA treatment significantly reduced the HFD- or PA-induced IL-1β secretion (Fig. 6(A,B)). These results demonstrate that SalA treatment inhibits activation of the NLRP3 inflammasome *in vivo* and *in vitro*.

**Figure 7:**
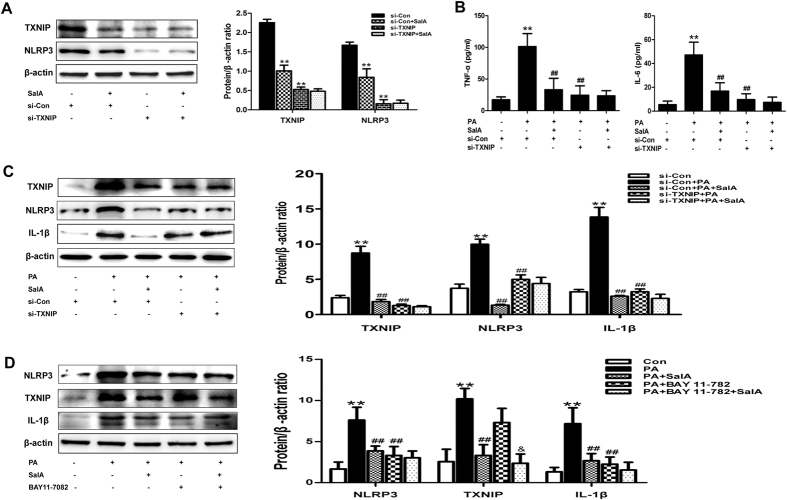
SalA inhibits NLRP3 inflammasome activation to prevent the release of pro-inflammatory cytokines in a TXNIP-dependent manner in PA-treated HepG2 cells. HepG2 cells were transfected with control siRNA or TXNIP siRNA for 48 h. Then, the transfected cells were treated with SalA (20 μM) for 6 h, and TXNIP and NLRP3 protein levels in the cellular lysate (**A**) were measured by Western blotting. The data are expressed as the mean ± SD (n = 3), ^**^P < 0.01 vs. the si-Con group; Furthermore, after pretreatment with SalA (20 μM), the transfected cells were exposed to PA (0.5 mM) for another 24 h. The protein expression of TXNIP, NLRP3 and IL-1β (**C**) was measured by Western blotting(n = 3), and the culture supernatant TNF-α and IL-6 levels (**B**) were measured by ELISA (n = 8). The data are expressed as the mean ± SD, ^**^P < 0.01 vs. the si-Con group, ^##^*P* < 0.01 vs. the si-Con + PA group. (**D**) HepG2 cells were pretreated with 20 μM SalA for 6 h and/or 15 μM BAY 11-7082 for 10 h before being exposed to PA (0.5 mM). The protein levels of NLRP3, TXNIP and IL-1β were evaluated by Western blotting. The data are expressed as the mean ± SD (n = 3), ^**^P < 0.01 vs. the control group, ^##^*P* < 0.01 vs. the PA group, ^&^*P* < 0.05 vs. the PA + BAY 11-7082 group.

Previous studies have suggested that TXNIP expression is closely associated with NLRP3 inflammasome activation^19^,^26^. We thus hypothesized that the inhibition of NLRP3 inflammasome activation by SalA was related to the down-regulation of TXNIP. We used a siRNA strategy to examine the role of TXNIP in the inhibition of NLRP3 inflammasome activation. As show in Fig. 7(A), compared with the control siRNA group, the group with SalA treatment exhibited down-regulation of both TXNIP and NLRP3 expression; furthermore, the siRNA-induced TXNIP knockdown nearly blocked NLRP3 expression, and SalA treatment no longer exerted any effects. These results suggest that TXNIP interacts with NLRP3 and that down-regulation of TXNIP may partially affect NLRP3 expression. Moreover, treatment with the specific NLRP3 inflammasome inhibitor BAY 11-7082^42^ had no effect on the PA-induced TXNIP overexpression (Fig. 7(D)), suggesting that TXNIP overexpression occurs upstream of hepatic NLRP3 inflammasome activation in response to PA exposure. Both SalA pretreatment and TXNIP silencing prevented the PA-induced overexpression of NLRP3 and IL-1β, as well as pro-inflammatory cytokine secretion. In contrast, pretreatment with SalA after TXNIP silencing had no such effect in PA-treated HepG2 cells (Fig. 7(B,C)). These results indicate that the protection conferred by SalA against HFD- or PA-induced hepatic inflammation is mediated by the TXNIP/NLRP3 inflammasome pathway.

### Inhibition of ChREBP nuclear translocation by SalA alleviates HFD-induced lipid accumulation: involvement of TXNIP

**Figure 8:**
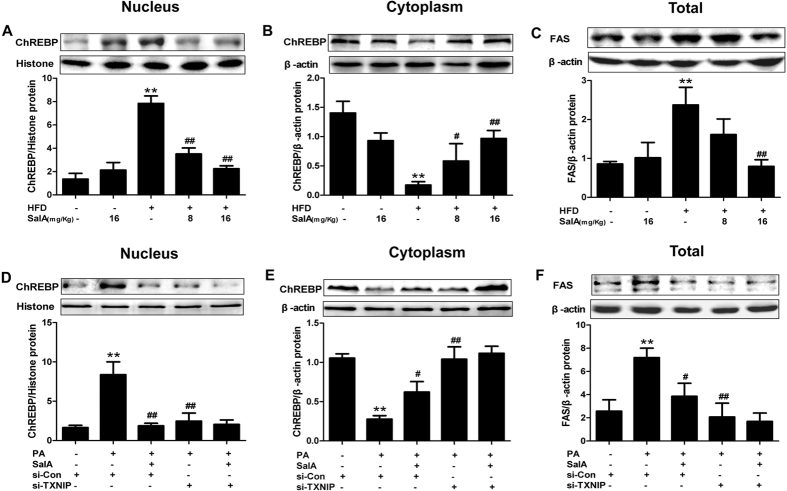
TXNIP mediates the inhibition of ChREBP nuclear translocation by SalA in PA-treated HepG2 cells. (**A,B**) The levels of nuclear and cytosolic ChREBP in rat livers were measured by Western blotting. (**C**) The total protein level of FAS in the rat liver was evaluated by Western blotting. The data are expressed as the mean ± SD (n = 3), ^**^P < 0.01 vs. the control group; ^#^*P* < 0.05, ^##^*P* < 0.01 vs. the HFD group. HepG2 cells were transfected with control siRNA or TXNIP siRNA for 48 h before treatment with SalA (20 μM). Then the transfected cells were exposed to PA (0.5 mM) for another 24 h. The levels of nuclear and cytosolic ChREBP in HepG2 cells were measured by Western blotting (**D,E**). Cellular lysate FAS protein levels were determined by Western blotting (**F**). The data are expressed as the mean ± SD (n = 3), ^**^P < 0.01 vs. control siRNA; ^#^*P* < 0.05, ^##^*P* < 0.01 vs. the si-Con + PA group.

The transcription factor ChREBP is intimately involved in hepatic lipogenesis, and nuclear translocation of ChREBP is an important process in the transcriptional regulation of lipogenic genes, including acetyl-CoA carboxylate (ACC) and fatty acid synthase (FAS)^33^,^43^,^44^. Our results showed that SalA-mediated correction of hepatic steatosis led to decreased liver and plasma levels of TG and NEFA (Fig. 2). We thus examined the possibility that SalA-mediated protection against NAFLD may involve the nuclear translocation of ChREBP. To identify a potential change in ChREBP translocation, the cytosolic and nuclear ChREBP contents were measured. Interestingly, although the ChREBP nuclear content was markedly increased in the HFD-fed group, a decreased cytosolic level of ChREBP was observed. In contrast, SalA treatment completely prevented the increase in the nuclear content of ChREBP (Fig. 8(A,B)). In parallel, the HFD-induced overexpression of FAS was decreased by SalA treatment (Fig. 8(C)). These results indicate that SalA regulates the abundance of nuclear ChREBP, presumably through the inhibition of its translocation from the cytosol to the nucleus.

TXNIP is reported to promote dephosphorylation and nuclear translocation of its transcription factor, ChREBP, resulting in a regulation of ChREBP target genes^36^. Moreover, we showed that SalA inhibited HFD- or PA-induced TXNIP overexpression (Fig. 5); thus, we investigated whether inhibition of TXNIP by SalA partially mediated the effects of SalA on the nuclear translocation of ChREBP, as well as the expression of its target genes in PA-treated HepG2 cells. In parallel with the above *in vivo* data, PA stimulation enhanced the cytosol-to-nucleus translocation of ChREBP and increased the FAS protein levels in HepG2 cells. Pretreatment with SalA completely inhibited this translocation of ChREBP and up-regulation of FAS, whereas the SalA-mediated inhibition was blocked by siRNA-mediated TXNIP knockdown (Fig. 8(D–F)). Taken together, these *in vivo* and *in vitro* results demonstrate that SalA ameliorates HFD-induced hepatic steatosis partially via regulation of the TXNIP/ChREBP pathway.

## Discussion

The pathogenic process of NAFLD is strongly linked to overnutrition; meanwhile, lipid accumulation, oxidative stress and inflammation have been widely suggested to play a pivotal role in the transition from steatosis to NASH^2^,^5^. Suppression of inflammation and oxidative stress via regulating signaling cascades implicated in hepatic inflammation and/or oxidative stress would make a great contribution to improving aspects of NAFLD^8^,^17^,^45^. However, effective therapeutic interventions against HFD-induced NAFLD provided by pharmacotherapy are currently limited. SalA, one of major phenolic compounds extracted from *Radix Salvia miltiorrhiza*, has been suggested to be a multi-target agent that mainly possesses anti-oxidative and anti-inflammatory properties^12^,^15^,^16^,^46^. In the present study, we found that SalA treatment effectively protected rats against HFD-induced liver injury, as indicated by following results: 1) SalA conferred protection against HFD- or PA-induced hepatic steatosis, inflammation and oxidative stress; and 2) the protective effects of SalA were associated with the TXNIP/NLRP3 and TXNIP/ChREBP pathways. Overall, the present study is the first to report that SalA exerts potent hepatoprotective functions in a HFD-fed rat model of NAFLD and to describe the mechanism by which SalA exerts its protective effects against HFD-induced NAFLD.

Recent emerging studies have indicated that TXNIP plays an important role in the pathogenesis of NAFLD and that targeting TXNIP may represent a therapeutic approach for treating steatohepatitis^24^. Oxidative stress has been considered a crucial contributor to the development of NAFLD^37^. As a key endogenous regulator of the cellular redox balance, TXNIP was recently reported to directly activate the NLRP3 inflammasome upon oxidative stress^26^,^32^. Activation of the NLRP3 inflammasome leads to tissue damage by causing the maturation and secretion of pro-inflammatory cytokines^28^. Meanwhile, TXNIP-induced NLRP3 inflammasome activation is involved in the pathogenesis of several metabolic liver diseases^8^,^19^. In the present study, our results showed that activation of the NLRP3 inflammasome occurred in parallel with the induction of oxidative stress, and the overexpression of TXNIP subsequently led to the secretion of pro-inflammation cytokines and inflammation in HFD-fed rats. Furthermore, TXNIP siRNA treatment prevented the PA-induced overexpression of NLRP3 and IL-1β in HepG2 cells, resulting in a decrease in the levels of pro-inflammatory cytokines, including TNF-α and IL-6 (Fig. 7). Moreover, treatment with a specific NLRP3 inflammasome inhibitor (BAY 11-7082) had no effects on the PA-induced TXNIP overexpression (Fig. 7(D)), suggesting that TXNIP overexpression occurs upstream of hepatic NLRP3 inflammasome activation in response to PA exposure. Therefore, the HFD-induced overexpression of TXNIP was related to the activation of the NLRP3 inflammasome and the inflammation observed during HFD-induced NAFLD.

On the basis of a previous study and our findings, we propose that targeting the TXNIP-NLRP3 inflammasome pathway may represent a therapeutic approach to attenuate liver injury during HFD-induced NAFLD. Moreover, some phenolic compounds have been reported to confer protective effects against NAFLD by targeting this pathway^8^,^19^,^41^,^47^. Likewise, in the present study, consistent with its effects on oxidative stress, SalA treatment effectively suppressed the overexpression of TXNIP and inhibited the activation of the NLRP3 inflammasome *in vivo* and *in vitro*. Moreover, the up-regulation of IL-1β and the higher levels of pro-inflammatory cytokines, such as TNF-α and IL-6, were decreased by SalA treatment. However, SalA treatment had no inhibitory effects on the PA-induced activation of the NLRP3 inflammasome and the pro-inflammatory cytokine secretion in TXNIP siRNA-transfected HepG2 cells. These results indicate that suppression of the TXNIP-NLRP3 inflammasome pathway is required for SalA to ameliorate hepatic inflammation and oxidative stress during HFD-induced NAFLD.

Recently, the transcription factor ChREBP has emerged as a key determinant in the control of hepatic lipogenesis, as well as in the pathogenesis of NAFLD^35^,^43^. Indeed, liver-specific inhibition of ChREBP improves hepatic steatosis and insulin resistance in obese ob/ob mice^43^. Moreover, ChREBP is overexpressed in patients with nonalcoholic steatohepatitis^34^. However, another study showed that ChREBP-mediated increases in the SCD1 (∆9-desaturase) activity in the liver may generate metabolically beneficial lipid species in mice fed an obesogenic HFD, resulting in an overall improvement of lipid homeostasis^48^. Thus, the molecular mechanism governing the control of ChREBP expression during NAFLD needs to be further elucidated. ChREBP activity is regulated by glucose metabolism and multiple post-translational modifications in the liver^49^,^50^,^51^. Upon activation, ChREBP translocates into the nucleus and binds to the ChoRE element present in the promoters of glycolytic and lipogenic genes, leading to hepatic lipid accumulation upon activation of lipogenic pathways^52^. As the cellular localization of ChREBP is a determinant of its functional activity, greater knowledge of the mechanisms involved in regulating its nucleo-cytoplasmic shuttling and/or its post-translational activation is crucial. Studies in pancreatic β-cells have shown that TXNIP transcription is mediated by a distinct ChoRE in the TXNIP promoter, which serves as the binding site for ChREBP^35^,^52^. TXNIP enhances ChREBP dephosphorylation and nuclear localization, resulting in a positive feedback loop, as well as the regulation of other ChREBP target genes. Moreover, TXNIP promotes ChREBP-mediated transcription partially through indirect inhibition of AMPK phosphorylation/activation^36^. Therefore, we speculated that the protective effect of SalA against HFD-induced hepatic lipid accumulation could be mediated, in part, through alterations in ChREBP nuclear translocation.

In the present study, we found that SalA altered ChREBP translocation from the cytosol to the nucleus. Moreover, TXNIP overexpression was found to occur in parallel with the up-regulation of the nuclear ChREBP protein levels *in vivo* and *in vitro*, which may cause up-regulation of FAS and hepatic lipid accumulation. In addition, TXNIP silencing markedly prevented ChREBP translocation from the cytosol to the nucleus (Fig. 8(D,E)). As expected, PA treatment-induced FAS overexpression was abrogated in TXNIP siRNA-transfected HepG2 cells (Fig. 8(F)). These results confirmed that the cross-talk mechanism between TXNIP and ChREBP partially contributes to the development of HFD-induced NAFLD. In addition, SalA effectively inhibited ChREBP nuclear translocation, whereas the TXNIP siRNA blocked such inhibition. Together, these results suggest that SalA-mediated protection against HFD-induced hepatic steatosis partly related to modulation of the hepatic TXNIP-ChREBP pathway. Furthermore, it should be mentioned that in recent studies, some polyphenols have been observed to play a role in inhibiting PPARγ expression and attenuating obesity in HFD-fed mice^53^; additionally, obesity, co-existing with NAFLD and sharing a common pathophysiology, has been considered an independent risk factor for NAFLD^54^,^55^. Considering these findings together with our results, we speculate that SalA might play a role in diet-induced obesity, and this effect may be associated with regulating the TXNIP-ChREBP pathway to a certain extent. However, the extent to which the anti-obesity effects of SalA exerted through regulating TXNIP-mediated signaling implicated in hepatic lipogenesis account for the overall improvement of NAFLD in HFD-fed rats remains to be further explored.

In summary, the present study is the first to demonstrate that SalA has a protective effect against HFD- or PA-induced NAFLD. The protective effect of SalA is associated with the down-regulated expression of TXNIP, which is accompanied by inhibition of NLRP3 inflammasome activation and ChREBP nuclear translocation, resulting in a dramatic reduction in hepatic pro-inflammation cytokines and lipid accumulation. Meanwhile, SalA treatment effectively attenuates the serious oxidative stress induced during NAFLD. These results indicate that SalA confers protection against NAFLD, at least partially by targeting hepatic TXNIP; however, further studies are needed to determine the precise underlying mechanism. The targeting of hepatic TXNIP by SalA may represent an attractive pharmacological target for the development of new drugs to impede the progression of NAFLD.

## Materials and Methods

### Experimental animals and reagents

Male Sprague-Dawley rats (180–220 g) were obtained from the Experimental Animal Center of Dalian Medical University (Dalian, China). The rats were kept in standard laboratory conditions with free access to food and water and were allowed to adapt to the new environment for one week before experimental procedures. All of the procedures were conducted according to the guidelines of the Institutional Animal Care and Use Committee of Dalian Medical University and were approved by the Institutional Ethics Committee of Dalian Medical University. SalA (98% purity) was purchased from Shanghai Winherb Medical Science Co., Ltd. (Shanghai, China) and dissolved in distilled water. The HFD contained several compounds that provide energy, as previously described^17^,^20^. After acclimation for one week, 40 experimental rats were randomly divided into 5 groups: control; control + SalA (16 mg/kg/d); HFD; HFD + SalA (8 mg/kg/d) and HFD + SalA (16 mg/kg/d). SalA was administered by gavage every day for 10 weeks. All the dosages were determined by preliminary experiments, and the control rats were treated with an equal volume of saline. At the end of the experiment, all animals were euthanized, and the liver, blood and other tissue samples were harvested for further analysis.

### Cell culture and treatment

The human hepatocellular carcinoma cell line HepG2 was cultured in Dulbecco’s modified Eagle’s medium (DMEM) that was supplemented with 10% (v/v) fetal bovine serum (FBS) (Science cell, no. 0500, USA). The cells were kept at 37 °C in a humidified incubator with 5% CO_2_. RES (98% purity) was purchased from Shanghai Winherb Medical Science Co., Ltd. (Shanghai, China), and PA (Sigma no. P-0500), FFA-free BSA (Sigma no. A-6003) and BAY 11-7082 (Sigma no. B5556) were purchased from Sigma-Aldrich (St. Louis, MO, USA). To establish the *in vitro* model of cellular fat accumulation, the cells were stimulated with 0.5 mM FFA/1% BSA for 24 h. A solution of 0.5 mM FFA/1% BSA was prepared as previously described^17^. After incubation with 20 μM SalA or 10 μM RES for 6 h or with 15 μM BAY 11-7082 for 10 h, the HepG2 cells were then subjected to 0.5 mM FFA/1% BSA stimulation as needed.

### Liver histologic examination

Liver tissues were fixed in 10% phosphate-buffered formalin and then paraffin-embedded. Tissue blocks were sectioned at a thickness of 4 μm and stained with hematoxylin-eosin reagent, then scored by a blinded histopathologist according to the NAFLD activity score (NAS) system^56^.

### Biochemical assays

Blood samples were obtained from the abdominal cavity and centrifuged at 3000 rpm for 15 min. Next, the serum was separated for further analysis. The serum levels of ALT, AST, TG, TC and NEFA were measured according to the manufacturer’s protocols. (Nanjing Jiancheng Corp., Nanjing, China). The TG, TC, NEFA, SOD, MDA and H_2_O_2_ levels in the liver were determined with commercial kits from Nanjing Jiancheng Bioengineering Institute (Nanjing, China) according to the manufacturer’s protocol. The final results were corrected for protein content.

### Measurement of cytokine levels

The levels of TNF-α and IL-6 in the serum and in the culture medium were measured using commercially available enzyme-linked immunosorbent assay (ELISA) kits from Cloud-Clone&USCN (Wuhan, China) according to the manufacturer’s instructions.

### Immunohistochemistry

Liver tissues were fixed in 10% phosphate-buffered formalin and then paraffin embedded. For immunohistochemical staining, tissue sections were deparaffinized with xylene and stepwise rehydrated with serial dilutions of ethanol. Antigen retrieval was performed by incubating the sections in antigen retrieval buffer (Beyotime Institute of Biotechnology, Shanghai, China) for 15 minutes at 97 °C. After antigen retrieval, the sections were incubated with 3% H_2_O_2_ for 15 min at room temperature, then with goat serum at 37 °C for 20 min and finally with F4/80 Ab (1:100 dilution, 18705-1-AP, Proteintech group, Wuhan, China) overnight at 4 °C. The sections were subsequently washed twice in phosphate buffered saline (PBS) and incubated with a biotinylated secondary antibody for 30 min at 37 °C. The sections were then washed again, incubated with streptavidin–peroxidase complex for 30 min at 37 °C, and washed once more in PBS. Colorization was monitored using a diaminobenzidine kit (SP-9001, ZSGB-BIO, Beijing, China). Finally, the sections were counterstained with hematoxylin, dehydrated and mounted with resinene (Beyotime Institute of Biotechnology, Shanghai, China) and examined microscopically (400×). From each animal, more than five tissue sections, including representative sections, were analyzed^57^.

### Determination of intracellular ROS and Nile Red staining

Cellular ROS were detected using the Reactive Oxygen Species Assay Kit (Beyotime Institute of Biotechnology, Shanghai, China) according to the manufacturer’s protocol. Cellular lipid accumulation was assessed by Nile Red (Sigma no. 19123) staining; Nile red was prepared as previously described^17^. The lipid-bound Nile Red fluorescence was observed with a fluorescence microscope.

### Western blotting

Total proteins and nuclear proteins were extracted from rat liver tissues using a protein extraction kit (KeyGEN Biotech, Nanjing, China), and cells were lysed with RIPA buffer. Equal amounts of protein from each sample were separated by 8–15% SDS-PAGE (Bio-Rad, Hercules, USA) and transferred to a polyvinylidene difluoride membrane (Millipore, Bedford, USA). Western blot analysis was performed with antibodies specific for TXNIP, NLRP3, ASC, caspase-1, IL-1β, FAS, histone-H3 (Proteintech group, Wuhan, China), ChREBP (Abcam, ab157153) and β-actin (ZSGB-BIO, Beijing, China). Nonspecific binding was blocked by incubation of the membranes with 5% skim milk for 2 h at 37 °C. Membranes were exposed to enhanced chemiluminescence plus reagents (Beyotime Institute of Biotechnology, Shanghai, China). Spectrophotometric analysis was performed with a BioSpectrum-410 multispectral imaging system and analyzed with a Gel-Pro Analyzer Version 4.0 (Media Cybernetics, MD, USA).

### RNA isolation and RT-PCR

Total RNA was isolated with TRIZOL reagent (TaKaRa, Dalian, China) according to the manufacturer’s instructions. The quantity and purity of the obtained total RNA samples were determined by UV spectroscopy (NanoDrop 2000 Spectrophotometer, Thermo Fisher Scientific, Waltham, MA, USA). The sequences of the primers used for the RT-PCR assay are shown in Supplementary Table S1. Two-step RT-PCR was carried out as described in the All-in-One First-Strand cDNA Synthesis SuperMix (AH321-01, TRANSGEN BIOTECH, BeiJing, China) for qPCR protocol. The reverse transcription conditions were as follows: 42 °C for 15 min, followed by 5 s at 85 °C for RT/RI inactivation. qPCR was performed using the following conditions: 30 s at 94 °C for denaturation; 5 s at 94 °C for annealing, and 30 s at 60 °C for extension.

### siRNA transfection and cell viability assay

HepG2 cells were transfected with a specific TXNIP siRNA (100 nM) or non-binding control siRNA (100 nM) using Lipofectamine 2000 (Invitrogen, Karlsruhe, Germany) according to the manufacturer’s instructions. The TXNIP siRNA sequences were as follows: sense 5′-CUCCCUGCUAUAUGGAUGUTT-3′ and antisense 5′-ACAUCCAUAUAGCAGGGAGTT-3′ (Genepharma, Shanghai, China). After transfection for 48 h, the cells were treated with 20 μM SalA for 6 h before being exposed to PA or not. The cells and culture medium were then collected for ELISA and Western blotting analysis. The cell viability was analyzed using a Cell Counting Kit (ZP328, Zoman Biotechnology Co., Ltd. Beijing, China). The experiment details were performed according to the manufacturer’s protocols.

### Statistical analyses

The results are expressed as the mean ± SD. Statistical analyses were performed using GraphPad Prism (version 5.0; GraphPad Prism Software, La Jolla, CA, USA). The data were analyzed with a two-tailed unpaired Student’s t-test or one-way analysis of variance to determine the statistical significance between the groups. Differences of P < 0.05 were considered significant.

## Additional Information

**How to cite this article**: Ding, C. *et al*. New insights into salvianolic acid A action: Regulation of the TXNIP/NLRP3 and TXNIP/ChREBP pathways ameliorates HFD-induced NAFLD in rats. *Sci. Rep.* **6**, 28734; doi: 10.1038/srep28734 (2016).

## Supplementary Material

Supplementary Information
