# Supplementary material for: New insights into salvianolic acid A action: Regulation of the TXNIP/NLRP3 and TXNIP/ChREBP pathways ameliorates HFD-induced NAFLD in rats
Source: Sci Rep. 2016 Jun 27;6:28734. doi: 10.1038/srep28734 (PMC4922017; doi:10.1038/srep28734)
Supplement: Supplementary Information [file srep28734-s1.pdf]

Supplementary information

New insights into salvianolic acid A action: Regulation of the TXNIP/NLRP3 and TXNIP/ChREBP pathways ameliorates HFD-induced NAFLD in rats

Chunchun Ding[1], Yan Zhao[1], Xue Shi[1], Ning Zhang[1], Guo Zu[2], Zhenlu Li[2], Junjun Zhou[1], Dongyan Gao[1], Li LV[1], Xiaofeng Tian[2], Jihong Yao[1]

[1]*Department of Pharmacology, Dalian Medical University, Dalian, 116044, China*

[2]*Department of General Surgery, Second Affiliated Hospital of Dalian Medical University, Dalian, 116023, China*

*Corresponding author

Dr. Jihong Yao

Department of Pharmacology

Dalian Medical University

Dalian, China

Email: yaojihong65@hotmail.com

Supplementary Figure 1.

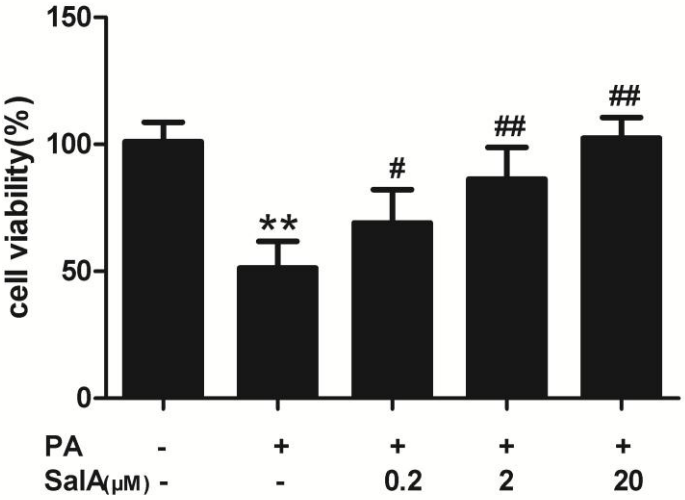

Supplementary Figure 1. **SalA protects HepG2 cells against PA-induced cytoxicity.** HepG2 cells were pretreated with SalA (0.2, 2, 20 μM) for 6 h and then subjected to 0.5 mM PA for 24 h. The viable cells were determined using the MTT reduction assay. Data are expressed as means ± SD. ** $P < 0.01$ vs. the control group, $^{\#}$ $P < 0.05$, $^{\#\#}$ $P < 0.01$ vs. the PA group (n = 8).

Supplementary Table 1.

Table 1

Primers for the RT-PCR assay.

Primer	Sequence
TXNIP	F:AAGCGTTGAGTAGTACAGATGAG
	R: ATGGCGTGGCAAGAGTC
NLRP3	F: AGCCTCAGGGCACCAA
	R: GGGATGAAGCACATAGTAAACA
β-actin	F: GGAAATCGTGCGTGACATTAAAG
	R: CGGCAGTGGCCATCTCTT
